# Special Issue: Chitin, Chitosan and Related Enzymes

**DOI:** 10.3390/molecules22071066

**Published:** 2017-06-27

**Authors:** Massimiliano Fenice

**Affiliations:** Laboratory of Microbiology, and Laboratory of Applied Marine Microbiology (Conisma), University of Tuscia, I-01100 Viterbo, Italy; fenice@unitus.it

Chitin and chitosan are very abundant natural polymers with distinctive properties, such as bioactivity, biocompatibility and biodegradability, that have inspired a number of basic and applied studies, mostly in biotechnology, medicine, food preservation and agriculture. The interest in these polysaccharides is still quite high and lot of scientists are currently devoting their efforts to improve the knowledge in this field, gather in various scientific societies spread all over the world and organise specific conferences. The same broad interest in chitin and chitosan has been devoted to the various enzymes involved in their hydrolysis and the corresponding enzyme-producing organisms, in particular bacteria and fungi.

Research on chitin, chitosan, and related enzymes has led to many basic and applied studies carried out in order to: explore the potential of these natural polymers (commercial products based on these polymers entered the market years ago), investigate the action and perspectives of the various enzymes and to directly apply the chitinolytic organisms. Although the field had been widely investigated, there is still room for interesting research novelties. 

This Special Issue aimed to provide a platform for the dissemination of the most advanced studies in this field and main topics were: the physics, chemistry, and biochemistry of chitin and chitosan; chitin, chitosan, and chitin-derivatives production; applications of chitin, chitosan, and their derivatives; and the bio-synthesis and bio-degradation of chitin and chitosan.

Various scientists participated in this issue dealing with these various topics, and the Editorial Committee sought to accept only peculiar and/or particularly innovative papers, including review papers, covering the various aspects of the issue as follows:

*Synthesis and Characterization of Glutamic-Chitosan Hydrogel for Copper and Nickel Removal from Wastewater* [1]*.* Chitosan preparations were treated with various concentrations of glutamic acid resulting in different types of glutamic-chitosan hydrogels that were tested for the removal of copper(II) and nickel(II) from wastewater. The increase of glutamic acid concentration led to a decrease of metal removal efficiency, probably due to a decrease in the pore size of the hydrogels.

*Inhibition of Listeria monocytogenes in Fresh Cheese Using Chitosan-Grafted Lactic Acid Packaging* [2]*.* Chitosan was grafted with d,l-lactic acid in aqueous media using *p*-toluensulfonic acid as catalyst to obtain a non-toxic, biodegradable packaging material that was characterized using scanning electron microscopy and other techniques. The grafting in chitosan with d,l-lactic acid produced films with improved mechanical properties. This material successfully extended the shelf life of fresh cheese and inhibited the growth of *Listeria monocytogenes* during 14 days at 4 °C and 22% relative humidity. The results were compared to control samples and commercial low-density polyethylene packaging demonstrating that the use of non-toxic chitosan-g-LA for packaging of fresh cheese offers a valid alternative for the preservation and conservation of this product and inhibited the growth of *Listeria monocytogenes*.

Chitosan Nanoparticles as Carriers for the Delivery of ΦKAZ14 Bacteriophage for Oral Biological Control of Colibacillosis in Chickens [3]. The use of chitosan as a delivery carrier has attracted much attention in recent years. In this study, chitosan nanoparticles and chitosan-ΦKAZ14 bacteriophage-loaded nanoparticles (C-ΦKAZ14 NP) were prepared, with the aim of achieving an effective protection of bacteriophage from gastric acids and enzymes in the chicken gastrointestinal tract. Chitosan nanoparticles showed considerable protection of the bacteriophage against enzymatic degradation, whereby the bacteriophage encapsulated in chitosan nanoparticles were protected, whereas the naked phagus was degraded. C-ΦKAZ14 NP was non-toxic as shown by a chorioallantoic membrane toxicity assay. The study demonstrate that chitosan nanoparticles could be a potent carrier of the tested bacteriophage for oral therapy against colibacillosis in poultry.

*Sorption of Cu(II) Ions on Chitosan-Zeolite X Composites: Impact of Gelling and Drying Conditions* [4]*.* Different typologies of chitosan-zeolite Na-X composite beads were prepared by encapsulation and analysed by a series of methods (i.e., X-ray diffraction, scanning electron microscopy, N_2_ adsorption–desorption and thermogravimetry) in order to investigate Cu(II) sorption. The drying method used for the storage of the adsorbent severely affected both the structural properties of the composite and the copper sorption effectiveness. Textural and Cu(II) sorption data indicated that whereas a chitosan coating impaired the accessibility of the microporosity of the zeolite; presence of zeolite improved the stability of chitosan dispersion upon supercritical drying and increased the affinity of the composites for Cu(II) cations.

The Psychrotolerant Antarctic Fungus Lecanicillium muscarium CCFEE 5003: A Powerful Producer of Cold-Tolerant Chitinolytic Enzymes [5]. This review paper summarizes the investigations carried out for more than 20 years on the Antarctic fungus Lecanicillium muscarium CCFEE 5003. This is a powerful producer of extracellular cold-tolerant enzymes being the production of chitin-hydrolyzing enzymes its principal extracellular catalytic activity. The chitinolytic system of L. muscarium is very complex and consists of a number of different proteins having various molecular weights and diverse biochemical characteristics, but their most significant trait is the marked cold-tolerance. Moreover, the fungus was able to exert a strong mycoparasitic action against various other fungi and oomycetes at low temperatures. Due to these characteristics, L. muscarium could play an important role in potential applications such as the degradation of chitin-rich materials at low temperature and the biocontrol of pathogenic organisms in cold environments. In view of future industrial application, the production of chitinolytic enzymes by the Antarctic fungus has been up-scaled and optimised in bench-top bioreactor.

*Chitosan and Its Derivatives as Highly Efficient Polymer Ligands* [6]*.* This review paper covers results of the last decade. Chitosan has a polyfunctional nature permitting its application as a polymer ligand not only for the recovery, separation, and concentration of metal ions, but also for the fabrication of various functional materials. Although unmodified chitosan itself is the unique cationic polysaccharide with very good complexing properties toward numerous metal ions, its sorption capacity and selectivity can be sufficiently increased and turned via chemical modification to meet requirements of the specific applications. The main information supplied by this paper was aimed to demonstrate how different strategies of chemical chitosan modification effect metal ions binding, and to underline the mechanisms involved in ions binding by chitosan derivatives.

All the published manuscripts have contributed to provide an image of the current and future trends in the development of chitin, chitosan and related enzyme research. The Editorial Committee expects that this Special Issue may help to understand the great potential of this specific research field and encourage further research on this matter.

## References

[1] Abdelwahab H.E., Hassan S.Y., Mostafa M.A., El Sadek M.M. (2016). Synthesis and Characterization of Glutamic-Chitosan Hydrogel for Copper and Nickel Removal from Wastewater. Molecules.

[2] Sandoval L.N., López M., Montes-Díaz E., Espadín A., Tecante A., Gimeno M., Shirai K. (2016). Inhibition of *Listeria monocytogenes* in Fresh Cheese Using Chitosan-Grafted Lactic Acid Packaging. Molecules.

[3] Kaikabo A.A., Sabo Mohammed A., Abas F. (2016). Chitosan Nanoparticles as Carriers for the Delivery of ΦKAZ14 Bacteriophage for Oral Biological Control of Colibacillosis in Chickens. Molecules.

[4] Djelad A., Morsli A., Robitzer M., Bengueddach A., di Renzo F., Quignard F. (2016). Sorption of Cu(II) Ions on Chitosan-Zeolite X Composites: Impact of Gelling and Drying Conditions. Molecules.

[5] Fenice M. (2016). The Psychrotolerant Antarctic Fungus *Lecanicillium muscarium* CCFEE 5003: A Powerful Producer of Cold-Tolerant Chitinolytic Enzymes. Molecules.

[6] Pestov A., Bratskaya S. (2016). Chitosan and Its Derivatives as Highly Efficient Polymer Ligands. Molecules.

